# Crizotinib vs platinum‐based chemotherapy as first‐line treatment for advanced non‐small cell lung cancer with different *ROS1* fusion variants

**DOI:** 10.1002/cam4.2984

**Published:** 2020-03-13

**Authors:** Haiyan Xu, Quan Zhang, Li Liang, Junling Li, Zhefeng Liu, Weihua Li, Lu Yang, Guangjian Yang, Fei Xu, Jianming Ying, Shucai Zhang, Yan Wang

**Affiliations:** ^1^ Department of Comprehensive Oncology National Cancer Center/National Clinical Research Center for Cancer/Cancer Hospital Chinese Academy of Medical Sciences Peking Union Medical College Beijing China; ^2^ Department of Medical Oncology Beijing Chest Hospital Capital Medical University Beijing China; ^3^ Cancer Chemotherapy and Radiation Department Peking University Third Hospital Beijing China; ^4^ Department of Medical Oncology National Cancer Center/National Clinical Research Center for Cancer/Cancer Hospital Chinese Academy of Medical Sciences Peking Union Medical College Beijing China; ^5^ Department of Medical Oncology Chinese PLA General Hospital Beijing China; ^6^ Department of Pathology National Cancer Center/National Clinical Research Center for Cancer/Cancer Hospital Chinese Academy of Medical Sciences Peking Union Medical College Beijing China

**Keywords:** crizotinib, efficacy, next‐generation sequencing, non‐small‐cell lung cancer, *ROS1*

## Abstract

**Background:**

*ROS1* gene fusion represents a specific subtype of non‐small cell lung cancer (NSCLC). Crizotinib is recommended for *ROS1‐*positive NSCLC due to its favorable outcome in published clinical trials. However, due to the low incidence of *ROS1‐*positive NSCLC, there is limited information on real‐world clinical outcomes in patients treated with either crizotinib or platinum‐based doublet chemotherapy.

**Methods:**

Outcomes were recorded in 102 patients with stage Ⅲb or Ⅳ NSCLC who were treated at four Chinese hospitals between April, 2010 and June, 2019.

**Results:**

Of the 102 patients followed, 71.6% were females, 81.4% were non‐smokers, and 98.0% had adenocarcinoma. First‐line treatment with crizotinib achieved a significantly longer median progression‐free survival (PFS) compared with platinum‐based chemotherapy (14.9 months vs 8.5 months, respectively; *P* < .001). Next‐generation sequencing (NGS) identified 61 patients who had *ROS1* fusion variants, including CD74 (n = 33) and non‐CD74 (n = 28) variants. In patients harboring CD74 fusion variants, the median PFS with first‐line crizotinib treatment was significantly longer than in those harboring non‐CD74 fusion variants (20.1 months vs 12.0 months, respectively; *P* = .046). However, in patients treated with platinum‐based chemotherapy, there was no significant difference in PFS between the CD74 and non‐CD74 variant groups (8.6 months vs 4.3 months, respectively; *P* = .115). Overall survival (OS) was not reached.

**Conclusions:**

First‐line therapy with crizotinib is more beneficial than platinum‐based chemotherapy in patients with advanced NSCLC with different *ROS1* fusion variants. Patients harboring CD74 fusion variants appear to respond better to crizotinib.

## Graphical abstract

For first‐line treatment in advanced NSCLC with *ROS1* rearrangement, crizotinib is more beneficial than platinum‐based chemotherapy. Patients harboring *ROS1* CD74 fusion variants who are treated with first‐line crizotinib therapy have better PFS than those harboring non‐CD74 fusion variants. With first‐line platinum‐based chemotherapy, patients in both the CD74 and non‐CD74 fusion variant groups demonstrated a short PFS with no significant difference observed.

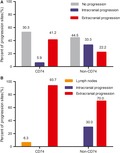

## 1 INTRODUCTION

*ROS1* gene fusion has become a new therapeutic target in patients with advanced or metastatic non‐small‐cell lung cancer (NSCLC) in addition to epidermal growth factor receptor (*EGFR*) mutations and anaplastic lymphoma kinase (*ALK*) gene fusions. The proportion of patients with *ROS1*‐positive NSCLC is lower than those of *EGFR‐*sensitive mutations and *ALK* gene fusions, with an overall prevalence of 1%‐2% in the US,^1^, ^2^, ^3^ and approximately 15 000 new cases of *ROS1‐*positive NSCLC annually.^4^ In an East Asian population, *ROS1* gene fusion has been identified in 2%‐3% of patients with NSCLC.^5^ Notably, the frequency accounted for 5.7% of patients with triple‐negative (*EGFR*, *ALK*, and *KRAS* wild‐type) NSCLC.^6^ *ROS1* gene fusion is observed predominantly in younger patients, in light smokers (less than 10 pack years), and/or those with a non‐smoking history who have adenocarcinoma.

*ROS1* gene fusion was first identified in a NSCLC cell line model, and was observed in a NSCLC patient's specimen by Rikova et al in 2007.^7^ In a preclinical study, *ROS1* gene fusions were shown to be associated with sensitivity to crizotinib therapy, and NSCLC patients harboring *ROS1* gene fusions have achieved partial responses to this agent. In an expansion cohort study of the PROFILE 1001 trial, 50 *ROS1*‐positive NSCLC patients demonstrated a high response rate (72%) and a median progression‐free survival (PFS) of 19.2 months when treated with crizotinib.^8^ Similarly, in an East Asian study, 127 *ROS1‐*positive NSCLC patients exhibited a response rate of 71.7% and a median PFS of 15.9 months.^9^

Currently, crizotinib is approved in many countries as a treatment option for patients with advanced NSCLC who harbor *ROS1* gene fusions, based on the results of clinical trials showing its effectiveness in such patients.^8^, ^10^, ^11^, ^12^ Although some studies have reported that patients with *ROS*1‐positive NSCLC who are treated with pemetrexed‐based chemotherapy also exhibit good efficacy in PFS,^13^, ^14^ these studies were single‐arm, retrospective analyses, and there is a lack of prospective, randomized, phase Ⅲ studies comparing crizotinib with platinum‐based chemotherapy as first‐line treatment for advanced NSCLC due to the rarity of *ROS1* rearrangements. In addition, studies reporting the outcomes of crizotinib treatment or platinum‐based doublet chemotherapy in patients with different *ROS1* gene fusions have not yet been identified.

Therefore, in this real‐world study, data on treatment outcomes were analyzed to compare crizotinib with platinum‐based doublet chemotherapy as first‐line treatment in *ROS1‐*positive NSCLC patients. We further analyzed disease progression patterns and survival outcomes among patients treated with crizotinib treatment and platinum‐based doublet chemotherapy who had different *ROS1* gene fusion variants.

## 2 METHODS

### 2.1 Patients

In a retrospective, multicenter study, 102 patients with stage IIIb or Ⅳ NSCLC who harbored *ROS1* gene fusions were treated at four hospitals in Beijing, China between 24 April 2010 and 6 January 2019. The flow chart of the study is shown in Data S1. *ROS1* gene fusion was confirmed by either the fluorescence in situ hybridization (FISH) detection method or by next‐generation sequencing (NGS) technology. Tissue samples originated from the lung (n = 69), lymph nodes (n = 22), pleural effusion (n = 6), and other sites (n = 5). Patients were confirmed as *ROS1*‐positive by FISH probe methods if there was a red and green split or an isolated signal in a kinase domain in at least 15% of tumor cells (after 50 tumor cells were counted in each sample).

All patients who met the following criteria were eligible: age ≥18 years; stage Ⅲb or Ⅳ NSCLC confirmed only *ROS1* gene fusions without other known mutation such as *EGFR*, *ALK*, *KRAS*, *MET* application, and *RET* fusion; recurrent or metastatic disease treated with crizotinib or platinum‐based doublet chemotherapy as first‐line therapy; and an Eastern Cooperative Oncology Group performance status (ECOG PS) score of 2 or less. Patients with brain metastases at baseline were also included. Patients must not have received any prior treatment for *ROS1* gene fusions other than crizotinib, or received synchronous chemoradiotherapy or immune‐directed therapy.

### 2.2 Treatment

Eligible patients were stratified into two groups on the basis of their treatment regimens. One group received crizotinib at a dosage of 250 mg twice every day. These patients’ disease was assessed approximately every 2 months. The other group received platinum‐based doublet chemotherapy for four or six cycles, followed by maintenance therapy which included bevacizumab combined with pemetrexed, or bevacizumab or pemetrexed alone every 21 days. These disease in those patients was assessed at baseline, after the first dose of treatment, and then after every two treatment cycles. Treatment regimens are summarized in Data S2. Some patients received carboplatin if they could not tolerate cisplatin. Eighteen patients received maintenance treatment with bevacizumab plus pemetrexed, or with pemetrexed or bevacizumab alone.

Treatment with crizotinib or chemotherapy was continued until either radiographic progressive disease (PD) or unacceptable toxicity developed.

### 2.3 Treatment assessments and definitions

Imaging examinations confirmed the measurable lesions documented by computed tomography (CT) scans of the chest and abdomen, magnetic resonance imaging (MRI) of the brain, or whole‐body bone scans, and the lesions were defined by Response Evaluation Criteria in Solid Tumors (RECIST), version 1.1. Tumor responses were assessed as either a complete response (CR), partial response (PR), stable disease (SD), or PD. The best response of each patient was recorded. The objective response rate (ORR) was defined as patients who showed a CR or PR, and the disease control rate (DCR) was defined as patients who showed a CR, PR, or SD.

The primary endpoint was PFS, which was measured from the first day of treatment initiation to the time of disease progression or death. Overall survival (OS) was calculated from the date of first‐line treatment to death or the last follow‐up. We also recorded the patterns of the first documented treatment failure. Central nervous system (CNS) progression was defined as intracranial failure. Extracranial progression was defined as distant organ metastases other than CNS metastases. Patients with combined extracranial and CNS metastases simultaneously were recorded only in the intracranial group. Patients harboring CD74 fusion variants and other fusion variants were assigned to the CD74‐*ROS1* fusion variant group. Smokers were defined as individuals who smoked currently or who reported a smoking history that included at least 100 cigarettes, while non‐smokers were defined as those with a smoking history of less than 100 cigarettes in their lifetime. Smoking history and ECOG performance status data were collected from electronic medical records, along with clinical information and survival outcome data. As this was a retrospective, non‐interventional study, it was exempted from obtaining patients’ informed consent. However, it was approved by an institutional ethics committee of the National Cancer Center/Cancer Hospital, Chinese Academy of Medical Sciences (approval 15‐144/1071).

### 2.4 Statistical analyses

Statistical analyses were performed using SPSS™ version 16.0 (SPSS Inc). Patient's characteristics at baseline were analyzed by applying descriptive statistics. The data were expressed as a percentage for dichotomous variables and analyzed using a Chi‐squared test or Fisher's exact test. The Kaplan‐Meier method was used to analyze the primary endpoint of PFS between groups. Univariate and multivariate analyses were performed using a Cox proportional hazard regression model, and all statistical tests were considered statistically significant if they were two‐tailed and *P* < .05. Kaplan‐Meier survival curves were created with GraphPad Prism 6.0.

## 3 RESULTS

### 3.1 Patients’ baseline characteristics

Of the 102 eligible patients, 73 (71.6%) were females and 29 (28.4%) were males. The median age at diagnosis of advanced NSCLC was 52 years (range 27‐82 years); 92 patients (90.2%) had a good PS score (0 or 1 point), and 83 (81.4%) were non‐smokers. One hundred patients (98.0%) were identified as having lung adenocarcinoma, and 16 presented with CNS metastases at baseline. 88 patients were diagnosed as stage IV, including 74 cases of initial diagnosis and 14 cases of recurrence. Almost half of the patients (n = 56; 54.9%) received oral crizotinib treatment as first‐line therapy, while the remaining 46 patients (45.1%) received a platinum‐based doublet chemotherapy regimen. The characteristics of the two treatment groups well balanced at baseline (Table 1).

**TABLE 1:** Baseline characteristics of 102 patients with *ROS1*‐positive advanced NSCLC

Characteristics	Total (n = 102)	Crizotinib (n = 56)	Chemotherapy (n = 46)	*P‐*value
Age, y (n, %)				
≥60	32 (31.4)	19 (33.9)	13 (28.3)	.539
<60	70 (68.6)	37 (66.1)	33 (71.7)	
Sex (n, %)				
Male	29 (28.4)	15 (26.8)	14 (30.4)	.684
Female	73 (71.6)	41 (73.2)	32 (69.6)	
Smoking history (n, %)				
Yes	19 (18.6)	8 (14.3)	11 (23.9)	.214
No	83 (81.4)	48 (85.7)	35 (76.1)	
Histological types (n, %)				
ADC	100 (98.0)	55 (98.2)	45 (97.8)	1.000
Non‐ADC	2 (2.0)	1 (1.8)	1 (2.2)	
Clinical stage (n, %)				
IIIb	14 (13.7)	5 (8.9)	9 (19.6)	.153
IV	88 (86.3)	51 (91.1)	37 (80.4)	
Recurrence	14 (15.9)	7 (13.7)	7 (18.9)	
ECOG score (n, %)				
0‐1	92 (90.2)	50 (89.3)	42 (91.3)	1.000
2	10 (9.8)	6 (10.7)	4 (8.7)	
Brain metastases at baseline (n, %)				
Yes	16 (15.7)	11 (19.6)	5 (10.9)	.280
No	86 (84.3)	45 (80.4)	41 (89.1)	
*ROS1* fusion subtype				
CD74	33 (32.3)	17 (30.4)	16 (34.8)	.503
Non‐CD74	28 (27.5)	18 (32.1)	10 (21.7)	
Unknown	41 (40.2)	21 (37.5)	20 (43.5)	

Abbreviations: ADC, adenocarcinoma; ECOG, Eastern Cooperative Oncology Group; NSCLC, non‐small cell lung cancer.

### 3.2 First‐line treatment outcomes with crizotinib and platinum‐based chemotherapy

Till the data cutoff date (June 30, 2019), 14 patients (13.7%) had died. The median follow‐up duration from the time of diagnosis to the data cutoff date was 24.9 months (range 6.0‐74.1 months). With first‐line oral crizotinib treatment, 47 patients achieved PR, seven had SD, and two had PD. In patients who received platinum‐based chemotherapy, CR was not observed in any patient, 26 had PR, 20 had SD. The ORR with first‐line crizotinib treatment was significantly better than with platinum‐based chemotherapy (83.9% vs 56.5%, respectively; *P* = .002), but significant difference was not observed in DCR between the two treatment groups (96.4% vs 100%, respectively; *P* = .195).

**FIGURE 1:**
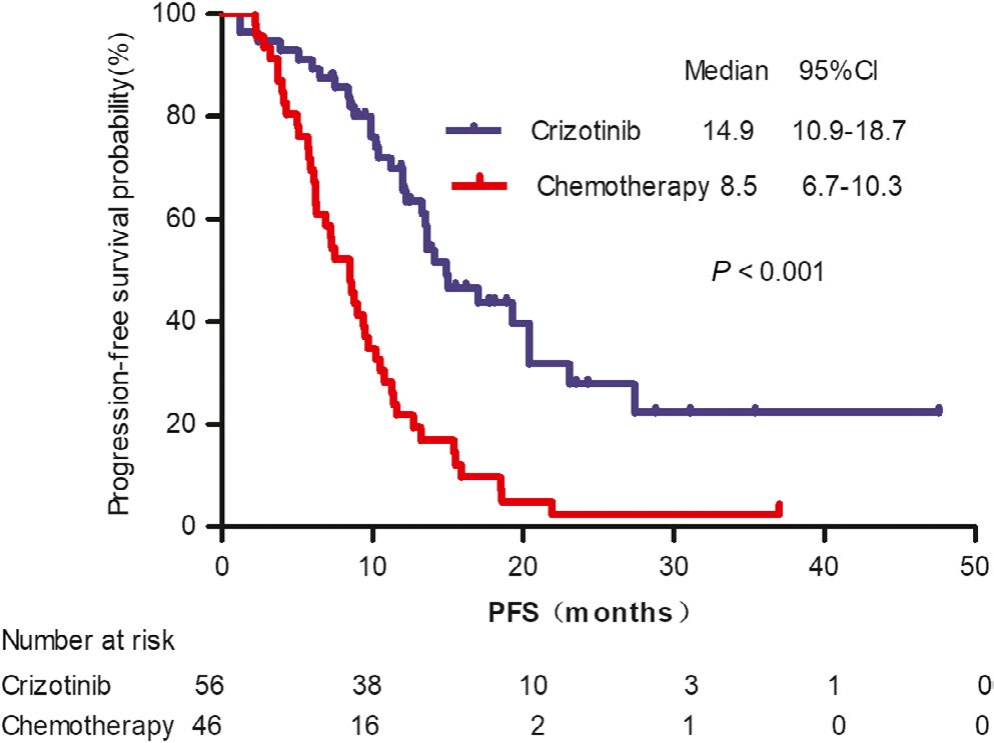
Kaplan‐Meier curves of progression‐free survival (PFS) with crizotinib treatment and platinum‐based chemotherapy in ROS1‐positive advanced non‐small cell lung cancer. The median PFS was significantly longer in patients treated with crizotinib compared with those treated with platinum‐based therapy (median 14.9 mo vs 8.5 mo, respectively; *P* < .001)

A total of 102 patients were divided into two groups including crizotinib treatment and platinum‐based doublet chemotherapy. The chemotherapy regimens were as follows: pemetrexed/platinum regimens (PP, n = 35), paclitaxel/platinum regimens (n = 5), docetaxel plus cisplatin(n = 2), and gemcitabine plus cisplatin (n = 4). The median PFS was significantly longer in patients who received crizotinib treatment in comparison with those who received platinum‐based doublet chemotherapy regimens (median 14.9 months, 95% CI 10.9‐18.7 months vs 8.5 months, 95% CI 6.8‐10.3 months, respectively; *P* < .001, Figure 1). We further analyzed the PFS in patients who received PP chemotherapy regimens (median 8.8 months, 95% CI 6.8‐10.8 months). Crizotinib treatment was also superior to PP chemotherapy regimens with significant difference observed between two groups (*P* < .001). OS was not reached in either treatment group.

### 3.3 *ROS1* fusion variants and disease progression patterns according to the different variants

NGS was performed on samples from 61 patients, and the *ROS1* fusion variants detected were CD74 (n = 30), SDC (n = 11), EZR (n = 7), SLC34A2 (n = 3), ZCCH (n = 2) and other variants (n = 5; one for each variant including SNN, KIAA1217, TFG, MYH9, and CCDC6). Three patients had dual fusion variants with a CD74 fusion variant and another fusion variant (MAGI1 & SLC25A26, CTXN3 & LNCO1184, and STXBP4 & HLF). Two patients had concurrent mutation (one patient has CD74 fusion with TP53 treated with crizotinib, another patient has SDC4 fusion with TP53 treated with chemotherapy). More than half of the *ROS1* gene fusion variants were CD74 variants (54.1%). Depending on whether the fusion variant included CD74, patients were divided into two groups: (a) those with CD74 variants and (b) those with non‐CD74 variants. The baseline characteristics of patients in these two groups were comparable (Table 2).

**TABLE 2:** Baseline characteristics of patients with the different *ROS1* fusion variants

Characteristics	Total (n = 61)	CD‐74 (n = 33)	Non‐CD74 (n = 28)	*P*‐value
Age, y (n, %)				
≥60	17 (27.9)	8 (24.2)	9 (32.1)	.493
<60	44 (72.1)	25 (75.8)	19 (67.9)	
Sex (n, %)				
Male	18 (29.5)	10 (30.3)	8 (28.6)	.883
Female	43 (70.5)	23 (69.7)	20 (71.4)	
Smoking history (n, %)				
Yes	12 (19.7)	7 (21.2)	5 (17.9)	1.000
No	49 (80.3)	26 (78.8)	23 (82.1)	
Histological types (n, %)				
ADC	59 (96.7)	31 (93.9)	28 (100)	.495
Non‐ADC	2 (3.3)	2 (6.1)	0 (0)	
Clinical stage (n, %)				
IIIb	10 (16.4)	6 (18.2)	4 (14.3)	.741
IV	51 (83.6)	27 (81.8)	24 (85.7)	
Recurrence	8 (15.7)	3 (11.1)	5 (20.8)	
ECOG scores (n, %)				
0‐1	53 (86.9)	29 (87.9)	24 (85.7)	1.000
2	8 (13.2)	4 (12.1)	4 (14.3)	
Brain metastases (n, %)				
Yes	9 (14.8)	2 (6.1)	7 (25.0)	.067
No	52 (85.2)	31 (93.9)	21 (75.0)	
First‐line therapy (n, %)				
Crizotinib	35 (57.4)	17 (51.5)	18 (64.3)	.315
Chemotherapy	26 (42.6)	16 (48.5)	10 (35.7)	

Abbreviations: ADC, adenocarcinoma; ECOG, Eastern Cooperative Oncology Group.

**FIGURE 2:**
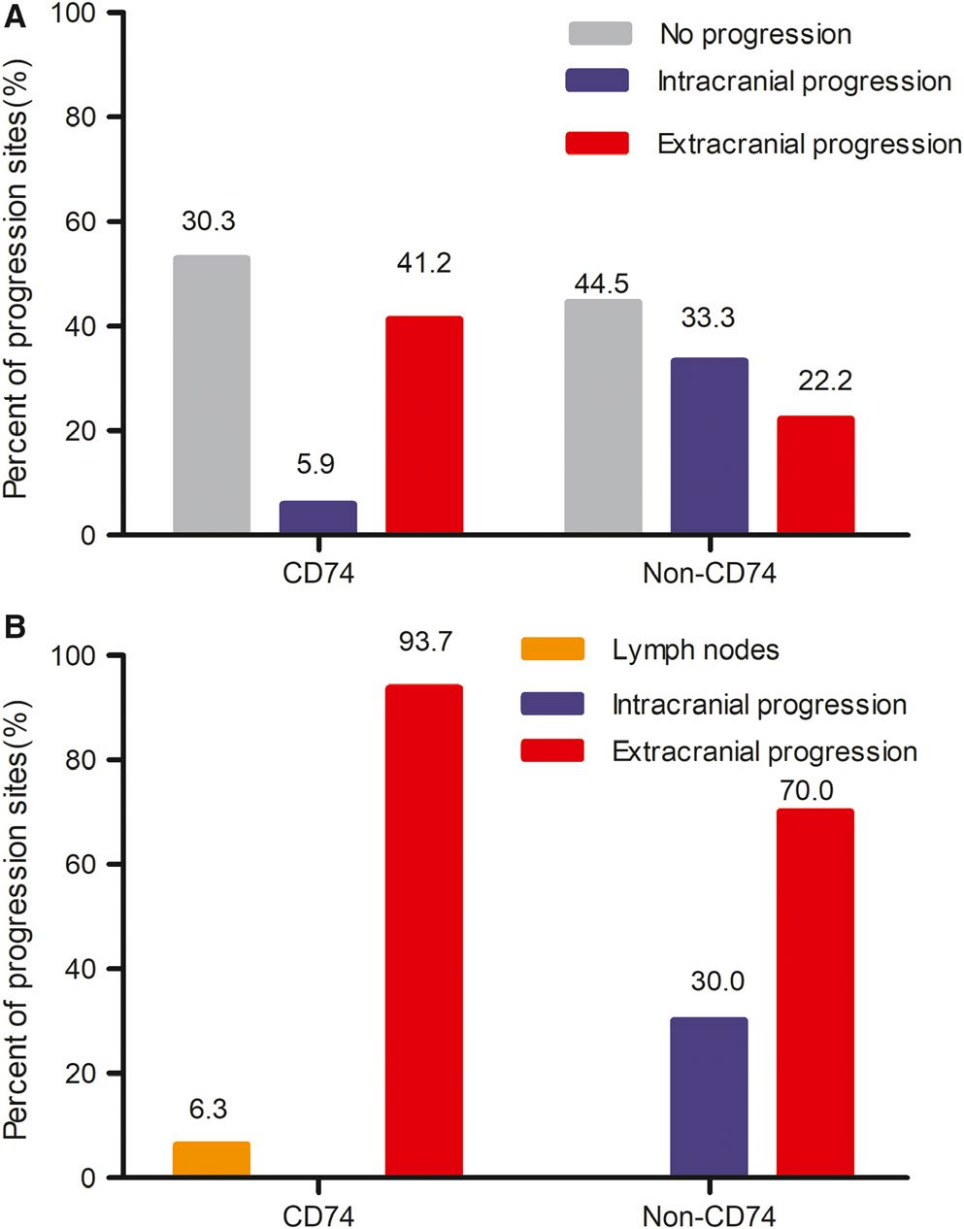
Disease progression patterns treated with crizotinib and platinum‐based therapy according to the different fusion variants. (A) With crizotinib treatment. (B) With platinum‐based chemotherapy

In *ROS1‐positive* NSCLC confirmed by NGS technology, 35 patients received crizotinib treatment and 26 received platinum‐based chemotherapy. At the data cutoff date, 18 patients exhibited disease progression in the crizotinib group, but all patients presented disease progression in the chemotherapy group. In the non‐CD74 fusion group, the proportion of patients with intracranial disease progression was higher than those in the CD74 group (33.3% vs 5.9%, respectively; Figure 2A). A total of seven patients treated with crizotinib had intracranial progression. There were six patients in the non‐CD74 group (three patients at baseline and three patients without brain metastases at initial diagnosis) and one patient in the CD74 group during the course of crizotinib treatment. The sites of disease progression in both two groups treated with platinum‐based chemotherapy were mainly extracranial progression (Figure 2B). All three patients who had intracranial progression were in the non‐CD74 group and no patient progressed in the CD74 group.

### 3.4 Survival analyses of crizotinib treatment or platinum‐based chemotherapy with different *ROS1* fusion variants

**FIGURE 3:**
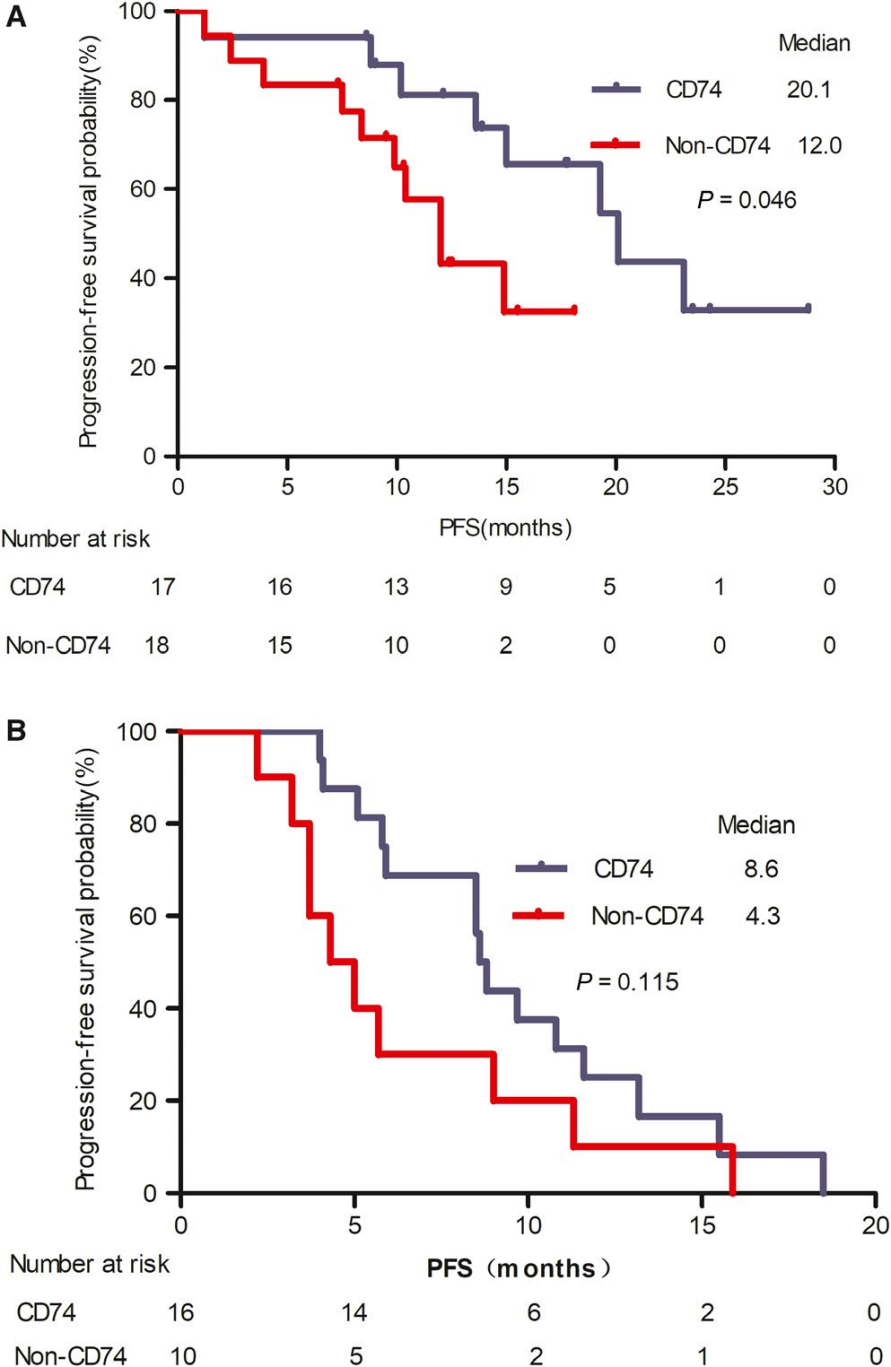
Kaplan‐Meier curves of progression‐free survival (PFS) with crizotinib treatment and platinum‐based chemotherapy for the different fusion variants. (A) With first‐line crizotinib treatment, the median PFS was significantly longer in patients harboring CD74 fusion variants in comparison with those harboring non‐CD74 fusion variants (20.1 mo; 95% CI 13.1‐27.0 vs 12.2 mo; 95% CI 9.1‐14.9, respectively; *P* = .046). (B) With first‐line platinum‐based chemotherapy treatment, no statistically significant difference in PFS was observed between patients with the different fusion variants (8.6 mo, 95% CI 8.1‐9.2 vs 4.3 mo, 95% CI 2.3‐6.3, respectively; *P* = .115)

To determine whether different *ROS1* gene fusion variants affect the therapeutic response, we analyzed the therapeutic effectiveness of crizotinib and platinum‐based chemotherapy as first‐line treatment with the different variants we identified, including 33 patients with CD74 fusion variants and 28 with non‐CD74 fusion variants. With first‐line crizotinib treatment, the median PFS was significantly longer in patients who harbored CD74 fusion variants than in those with non‐CD74 fusion variants (20.1 months, 95%CI 13.1‐27.0 months vs 12.0 months, 95% CI 9.0‐14.9 months, respectively; *P* = .046) (Figure 3A). However, no significant difference in PFS between the two groups was observed when platinum‐based chemotherapy was used in first‐line (8.6 months, 95% CI 8.1‐9.2 months vs 4.3 months, 95% CI 2.3‐6.3 months, respectively; *P* = .115) (Figure 3B). The median OS was not reached in either group.

### 3.5 Univariate and multivariate analyses for PFS by the Cox regression model

Univariate analysis demonstrated that the PFS in *ROS1*‐positive NSCLC patients was significantly associated with treatment patterns (crizotinib vs chemotherapy, *P* < .001) (Table 3). Although there was no statistically significant difference in PFS with brain metastases due to the small sample size, the PFS with crizotinib had shorter PFS in patients with brain metastases than in those without brain metastases (12.0 months vs 15.0 months, *P* = .249). The status of brain metastases and *ROS1* fusion subtype might affect the outcome of PFS. Therefore, the status of brain metastases, *ROS1* fusion subtype, and treatment patterns were entered into the multivariate Cox regression model. Multivariate analyses confirmed that *ROS1* fusion subtype and treatment pattern were independent predictors of PFS in *ROS1*‐positive NSCLC patients (*P* < .05, Table 4).

**TABLE 3:** Univariate survival analyses for PFS

Variable	B	SE	HR	95% CI	*P*
Age (≥60 vs <60)	−0.008	0.255	0.992	0.602‐1.635	.976
Gender (male vs female)	0.006	0.257	1.006	0.608‐1.663	.982
Smoking history (yes vs no)	0.103	0.290	1.109	0.629‐1.956	.722
Histological types (ADC vs Non‐ADC)	−0.948	0.724	0.388	0.094‐1.603	.291
Clinical stage (IIIb vs IV)	−0.161	0.341	0.851	0.437‐1.661	.637
ECOG score (0‐1 vs 2 points)	−0.056	0.374	0.946	0.454‐1.970	.882
Brain metastases at baseline (yes vs no)	0.123	0.306	1.131	0.622‐2.059	.686
Fusion subtype (CD74 vs Non‐CD74)	−0.532	0.311	0.587	0.319‐1.082	.088
Treatment (crizotinib vs chemotherapy)	−1.133	0.238	0.322	0.202‐0.513	<.001

Abbreviations: ADC, adenocarcinoma; CI, confidence interval; ECOG, Eastern Cooperative Oncology Group; HR, hazard ratio; PFS, progression‐free survival.

**TABLE 4:** Predictors of PFS analyzed by multivariate Cox regression

Variable	B	SE	HR	95% CI	*P*
Brain metastases at baseline (Yes vs No)	0.088	0.325	1.092	0.578‐2.065	.786
Fusion subtype (CD74 vs Non‐CD74)	−0.669	0.327	0.512	0.270‐0.972	.041
Treatment (Crizotinib vs Chemotherapy)	−1.217	0.241	0.296	0.185‐0.475	<.001

Abbreviations: CI, confidence interval; HR, hazard ratio; PFS, progression‐free survival.

## 4 DISCUSSION

Targeted therapies have been recommended as first‐line treatment in patients with advanced, recurrent, or metastatic *ROS1*‐positive NSCLC, as well as for *EGFR* mutations or *ALK* gene fusions. In this study, we analyzed the clinicopathological characteristics and disease progression patterns of NSCLC patients with different *ROS1* gene fusion variants who received first‐line treatment with either crizotinib or platinum‐based chemotherapy. Similar to the findings of previous studies,^6^, ^8^, ^15^, ^16^, ^17^, ^18^ we found that *ROS1* gene fusions occurred predominately in younger patients, women, and patients with adenocarcinoma without smoking history. At the time of the initial diagnosis, the incidence of brain metastases in the *ROS1*‐positive NSCLC patients we studied was only 15.7%, which is lower than that of patients with *ALK‐*rearranged NSCLC.^19^, ^20^, ^21^ For first‐line therapy, in patients treated with crizotinib treatment, disease progression in the brain showed a significant difference between the different *ROS1* fusion variants (*P* < .05); 33.3% of patients in the non‐CD74 variant group had intracranial progression, as compared with only 5.9% of those in the CD74 variant group. In patients treated with platinum‐based chemotherapy, the sites of disease progression were mainly extracranial progression. A reasonable explanation for this finding is that the proportion of patients with brain metastases at baseline treated with crizotinib was higher in the non‐CD74 variant group than those in the CD74 group (27.7% vs 11.8%, respectively). Another reason may be that patients in the non‐CD74 group are prone to intracranial progression. In addition, permeability of crizotinib through the blood–brain barrier may have resulted in higher incidence of brain metastasis.^22^, ^23^ In this regard, studies by Kaneda et al^24^ and Metro et al^22^ have reported that ratio of crizotinib in cerebrospinal fluid to plasma was very low (only 0.0026 to 0.006).

Our study also provided outcome data for both crizotinib and platinum‐based chemotherapy given as first‐line treatment in patients with different *ROS1* fusion variants. In comparison with platinum‐based chemotherapy, crizotinib treatment achieved significantly higher ORR (83.9% vs 56.5%, respectively; *P* = .002), and PFS (14.9 months vs 8.5 months, respectively; *P* < .001). The clinical benefit of crizotinib was consistent with those reported by Wu et al^9^ and Zeng et al^25^ in *ROS1‐*positive NSCLC patients in the East Asian population. Univariate and multivariate analyses confirmed that the PFS in *ROS1*‐positive NSCLC patients was significantly associated with treatment patterns, and crizotinib treatment was more beneficial than chemotherapy. However, several studies have found that *ROS1*‐positive NSCLC patients appeared to be sensitive to pemetrexed‐based treatment.^26^, ^27^ Drilon et al^28^ reported that *ROS1*‐positive patients who were treated with pemetrexed‐based chemotherapy had good ORR (78%) and long PFS (23 months), which were higher than our study. A possible reason was that 70% of the patients in the study of Drilon et al^28^ received maintenance therapy as compared with only 39.1% of patients (18/46) in our study. Another reason may be the differences between eastern and western populations. Although pemetrexed‐based chemotherapy has shown an effective clinical response rate in *ROS1*‐positive NSCLC patients, it is less efficacious than crizotinib treatment. It is not safe to conclude that platinum‐based chemotherapy was more beneficial than crizotinib treatment for *ROS1*‐positive advanced NSCLC based on single‐arm, small‐sample size study.

The traditional approach to detect *ROS1* gene fusion is FISH test, which is the gold standard, but the FISH test does not provide detailed information on *ROS1* gene fusion variants. With the development of comprehensive molecular profiling of NSCLC, NGS can detect new fusion variants and more information regarding the fusion variants. Few studies have reported therapeutic outcomes with crizotinib as first‐line treatment for patients with different *ROS1* fusion variants. In our study, patients harboring CD74 fusion variants treated with crizotinib treatment tended to have a longer PFS than those harboring non‐CD74 fusion variants (median 20.1 months vs 12.0 months, respectively). Median PFS extended for 8.1 months, and it was significant for the management of advanced or metastatic NSCLC. The finding reported by Michels et al^29^ indicates that first‐line crizotinib treatment may be more beneficial in patients with CD74 *ROS1* fusion variants. Multivariate analyses further demonstrated that *ROS1* fusion subtype was a valuable predictor of PFS in *ROS1*‐positive NSCLC patients. While the finding reported by Li et al^30^ showed that the median PFS with crizotinib treatment in the CD74 variant group was shorter than that reported in our study. The reasons for these potential discrepancies in results between our data and previous study might be explained by demographic or baseline characteristics. Notably, in our study, 2 patients (11.8%) in the CD74 variant group had a lower incidence of brain metastases at baseline than those in the non‐CD74 variant group (n = 5, 27.7%). All six (16.7%) patients who had brain metastases were in the CD74 group and no patient had brain metastases in non‐CD74 group in Li et al study.^30^ The PFS of patients treated with crizotinib in the non‐CD74 group was probably shorten by the short PFS in those with brain metastases. Although the status of brain metastases had no statistically significant effect on PFS due to the small sample size, short PFS in our study was founded in patients with brain metastases. A larger‐scale study on brain metastases is warranted. With platinum‐based doublet chemotherapy, patients in both two groups demonstrated short PFS with no significant difference between them.

As our study was a retrospective analysis, there might have some selection bias. In addition, several other limitations must be noted. First, the sample size may have been inadequate due to the low occurrence rate of *ROS1* gene fusions. Second, in the CD74 variant group, three patients were found to have *ROS1* double fusion variants using NGS, but we did not analyze how these patients responded to crizotinib treatment or to chemotherapy separately for the reason of small number. Third, disease assessments in the two study cohorts were evaluated in different frequency, which might cause PFS bias. However, it was speculated that there was little influence on the results due to significant differences in PFS. Lastly, comparison between patients treated with different lines of crizotinib and chemotherapy was not performed due to the immature nature of the data.

## 5 CONCLUSIONS

The data from the present study indicate that crizotinib is more beneficial than platinum‐based chemotherapy as first‐line therapy for *ROS1*‐positive NSCLC. Also, *ROS1* gene fusion subtype might be a predictive biomarker for advanced NSCLC. In *ROS1*‐positive NSCLC patients who harbored CD74 gene fusion variants, crizotinib tended to be more effective than in those who harbored non‐CD74 fusion variants.

## CONFLICT OF INTEREST

None of the authors has any no conflict of interest to disclose.

## AUTHOR CONTRIBUTIONS

All authors can take responsibility for the integrity of the data and the accuracy of the data analysis. Haiyan Xu, Quan Zhang, and Li Liang contributed to concept and design. Junling Li, Zhefeng Liu, Weihua Li, Guangjian Yang, Jianming Ying, and Shucai Zhang contributed to acquisition, analysis, or interpretation. Haiyan Xu, Lu Yang, Fei Xu, and Yan Wang contributed to discussion and revision. All authors read and approved the final manuscript.

## Supporting information

Data S1

Data S2

## Data Availability

The material supporting the conclusion of this study has been included in the article. Some information was provided in the supplementary files, and all other relevant data are available from the corresponding authors of this study.
