# Supplementary figures and images for: Crizotinib vs platinum‐based chemotherapy as first‐line treatment for advanced non‐small cell lung cancer with different ROS1 fusion variants
Source: Cancer Med. 2020 Mar 13;9(10):3328–36. doi: 10.1002/cam4.2984 (PMC7221311; doi:10.1002/cam4.2984)

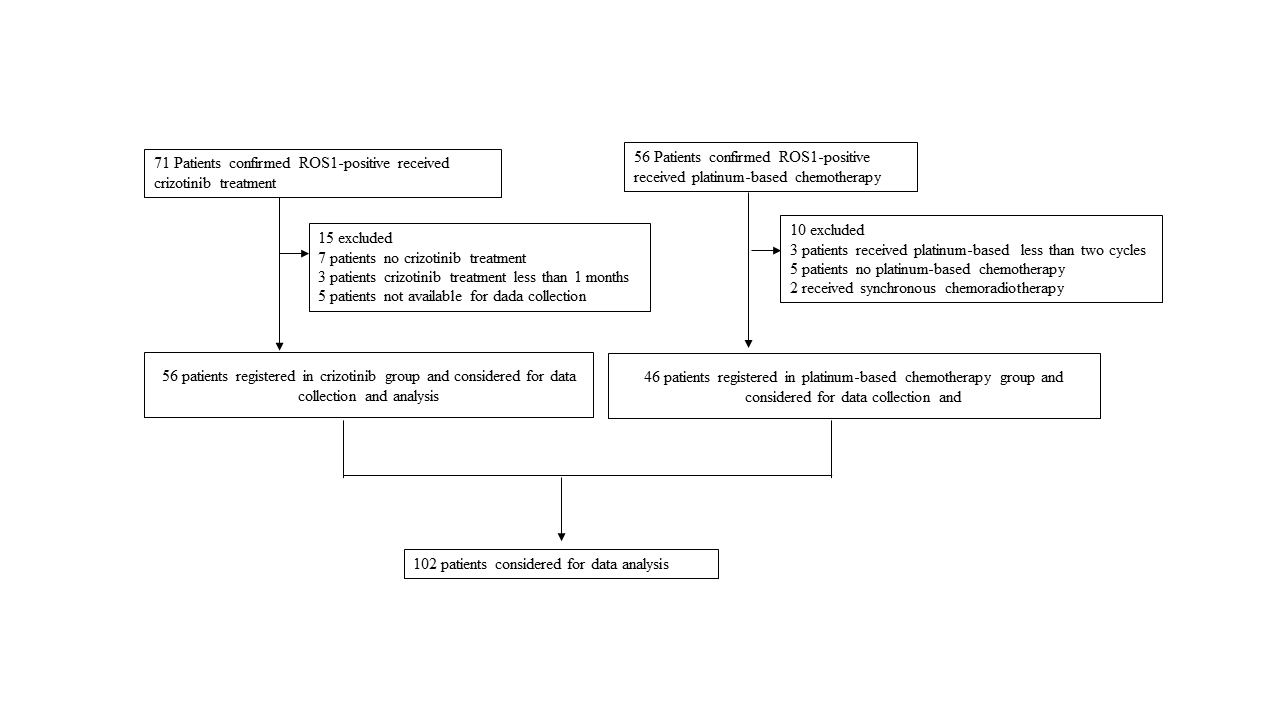

Supplement: Supplementary file 1 — Data S1 [file CAM4-9-3328-s001.tif]
