# Supplementary material for: Crizotinib vs platinum‐based chemotherapy as first‐line treatment for advanced non‐small cell lung cancer with different ROS1 fusion variants
Source: Cancer Med. 2020 Mar 13;9(10):3328–36. doi: 10.1002/cam4.2984 (PMC7221311; doi:10.1002/cam4.2984)
Supplement: Supplementary file 2 — Data S2 [file CAM4-9-3328-s002.docx]

Suppl 2. Platinum-based treatment regimens

| Regimens | Dose | Maintenance treatment |
| --- | --- | --- |
| Pemetrexed plus cisplatin or carboplatin (n=35) | Pemetrexed,500mg/m^2^intravenously on day 1  cisplatin,75 mg/m^2^ given over three days (d1-3)  or carboplatin, AUC=5 on day 2  3-wk cycle | Bevacizumab,7.5 mg/kg on day 1 (n=3)  or pemetrexed 500mg/ m^2^ on day 1 (n= 7)  or bevacizumab ,7.5 mg/kg on day 1 plus pemetrexed 500mg/ m^2^on day 1 (n=5) |
| Paclitaxel plus cisplatin or carboplatin (n=5) | Paclitaxel,175 mg/m^2^ on day 1  cisplatin,75 mg/m^2^ given over three days (d1-3)  or carboplatin, AUC=5 on day 2  3-wk cycle | Bevacizumab,7.5 mg/kg on day 1(n=2) |
| Docetaxel plus cisplatin  (n=2) | Docetaxel,75 mg/m^2^ on day 1  cisplatin,75 mg/m^2^ given over three days(d1-3)  3-wk cycle | Bevacizumab,7.5 mg/kg on day 1 (n=1) |
| Gemcitabine plus cisplatin (n=4) | Gemcitabine,1000 mg/m^2^ on day 1 and day 8  cisplatin 75 mg/m^2^ given over three days (d1-3)  3-wk cycle | No |

| **Tables 2.** Baseline characteristics of patients with the different *ROS1* fusion variants | | | | |
| --- | --- | --- | --- | --- |
| Characteristics | Total  (n = 61) | CD-74  (n = 33) | Non-CD74  (n = 28) | *P*-value |
| Age, years (n, %):  ≥60  <60 | 17 (27.9)  44 (72.1) | 8 (24.2)  25 (75.8) | 9 (32.1)  19 (67.9) | 0.493 |
| Sex (n, %):  Male  Female | 18 (29.5)  43 (70.5) | 10 (30.3)  23 (69.7) | 8 (28.6)  20 (71.4) | 0.597 |
| Smoking history (n, %):  Yes  No | 12 (19.7)  49 (80.3) | 7 (21.2)  26 (78.8) | 5 (17.9)  23 (82.1) | 1.000 |
| Histological types (n, %):  ADC  Non-ADC | 59 (96.7)  2 (3.3) | 31 (93.9)  2 (6.1) | 28 (100)  0 (0) | 0.493 |
| Clinical stage (n, %):  IIIB  IV | 13 (21.3)  48 (78.7) | 8 (24.2)  25 (75.8) | 5 (17.9)  23 (82.1) | 0.755 |
| ECOG scores (n, %)  0-1  2 | 54 (88.5)  7 (11.5) | 30 (90.9)  3 (9.1) | 24 (85.7)  4 (14.3) | 0.693 |
| Brain metastases (n, %):  Yes  No | 9 (14.8)  52 (85.2) | 2 (6.1)  31 (93.9) | 7 (25.0)  21 (75.0) | 0.067 |
| First-line therapy (n, %):  Crizotinib  Chemotherapy | 35 (57.4)  26 (42.6) | 17 (51.5)  16 (48.5) | 18 (64.3)  10 (35.7) | 0.315 |
| *Abbreviations:* ADC, adenocarcinoma; ECOG, Eastern Cooperative Oncology Group. | | | | |
